# Stage 4 Non-small Cell Lung Cancer With Human Epidermal Growth Factor Receptor 2 Alterations and Myocarditis Induced by Immune Checkpoint Inhibitors: A Case Report

**DOI:** 10.7759/cureus.48859

**Published:** 2023-11-15

**Authors:** Rakan Abulnaja

**Affiliations:** 1 Internal Medicine, Faculty of Medicine King Abdulaziz University Hospital, Jeddah, SAU; 2 Oncology, King Abdulaziz University Hospital, Jeddah, SAU

**Keywords:** targeted anticancer therapy, pembrolizumab side effect, non-small cell lung carcinoma (nsclc), drug-induced myocarditis, immune-checkpoint inhibitor (ici), human epidermal growth factor receptor 2 (her2)

## Abstract

Immune checkpoint inhibitor (ICI)-induced myocarditis is one of the most serious and potentially fatal toxicities of immunotherapy. Most of the guidelines for managing this toxicity are based on expert opinions. Human epidermal growth factor receptor 2 (HER2) alterations in non-small cell lung cancer (NSCLC) could be found using next-generation sequencing (NGS) on tissue and liquid biopsies. There is an approved first-line targeted therapy for HER2-positive breast and gastroesophageal cancers. Until now, no first-line targeted therapy for NSCLC with HER2 alterations has been approved. This case report presents a patient with metastatic HER2 NSCLC with a high PD-L1 level. She was started on first-line single-agent immunotherapy pembrolizumab. She tolerated the first two cycles well. Before the third cycle, she had palpitations and was tachycardiac. Furthermore, investigations found raised troponin levels. She was diagnosed with ICI-induced myocarditis. After being admitted to the cardiac care unit (CCU) and beginning pulse steroid treatment, she responded well with decreasing troponin levels.

## Introduction

The most commonly used immunotherapy for cancer treatment is immune checkpoint inhibitor (ICI). There are many types of ICI, such as anti-PD-1 (nivolumab, pembrolizumab, and cemiplimab), anti-PD-L1 (atezolizumab, avelumab, and durvalumab), and anti-CTLA-4 (ipilimumab and tremelimumab). Immunotherapy is generally better tolerated than chemotherapy, which possesses a distinct toxicity profile and mechanism. Immune-related adverse events (irAEs) can affect any organ. The most common irAEs include dermatitis, myositis, colitis, and endocrinopathies, with myocarditis being among the most serious.

HER2 alterations in advanced or metastatic non-small cell lung cancer (NSCLC) can be identified through next-generation sequencing (NGS), either on tissue or liquid biopsy. Unfortunately, unlike breast and gastroesophageal cancers, which have approved first-line targeted therapies (trastuzumab in both cancers and pertuzumab in breast cancer), there has not yet been an approved targeted therapy for the first line of treatment in lung cancer. The first-line treatment for lung cancer, depending on the PD-L1 value, is immunotherapy, with or without chemotherapy.

Trastuzumab is a monoclonal antibody that targets HER2, also known as ErbB2. It is primarily used in treatment for cancers overexpressing HER2, especially breast cancer.

## Case presentation

A 73-year-old female first visited the oncology clinic on December 7, 2022. In September 2022, she presented with a chronic dry cough and was initially treated for an upper respiratory tract infection and gastroesophageal reflux disease. However, the cough persisted, leading to further investigations. She has a medical history of osteopenia and has undergone a tonsillectomy. She reports no known allergies. For osteopenia, she has been taking vitamin D, calcium, and risedronic acid. Her family history is significant, with her maternal aunt and paternal grandmother both having had breast cancer. Additionally, she is an ex-smoker who quit 39 years ago, with a history of 15 packs/years.

**Figure 1:**
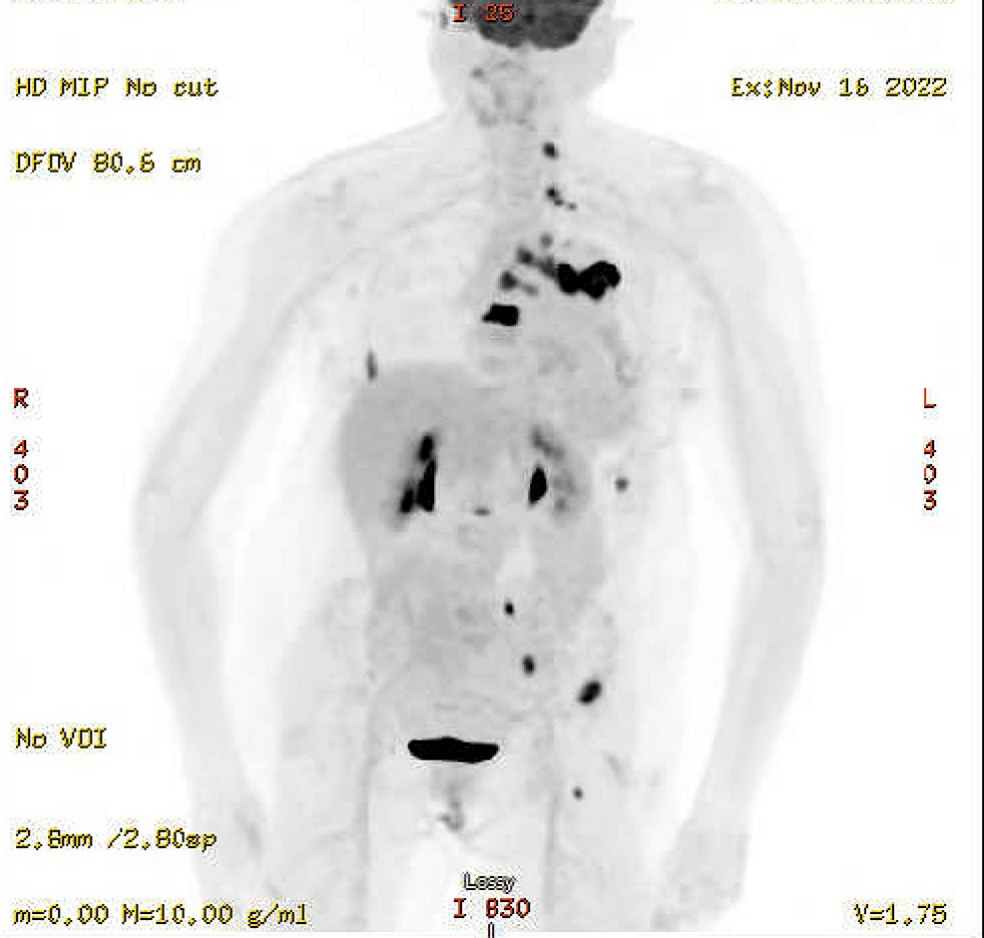
FDG-PET prior to immunotherapy. FDG-PET: Fluorodeoxyglucose-Positron Emission Tomography.

**Figure 2:**
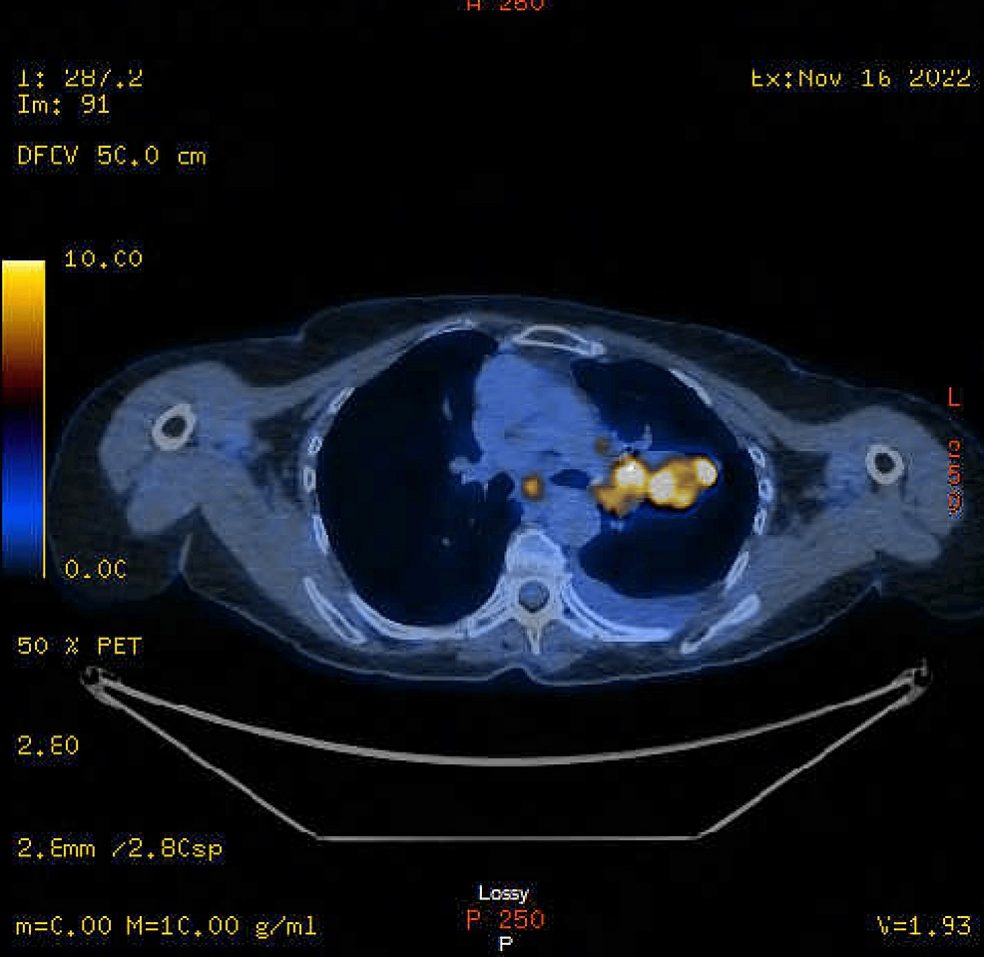
FDG-PET showing the left lung mass. FDG-PET: Fluorodeoxyglucose Positron Emission Tomography.

**Figure 3:**
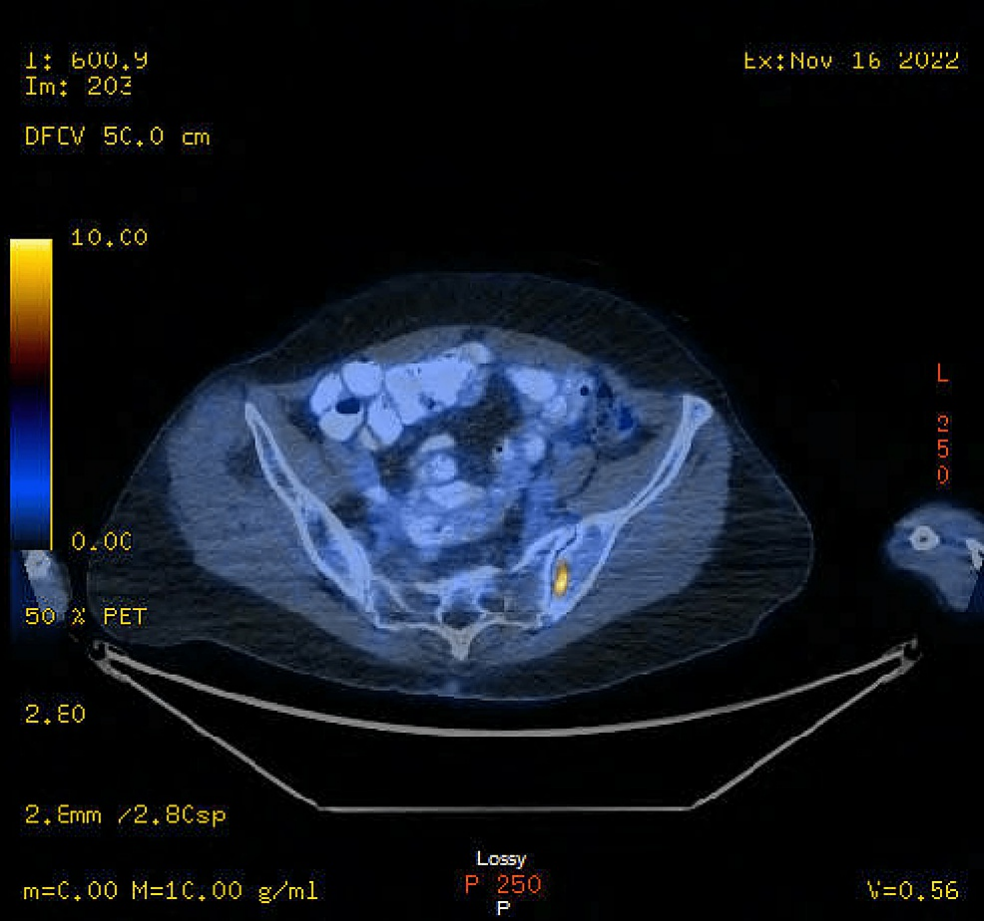
FDG-PET showing left femur involvement. FDG-PET: Fluorodeoxyglucose Positron Emission Tomography.

The patient had a normal chest X-ray in October 2022. She also underwent a CT scan of the chest on October 21, 2022, that showed a left perihilar mass and mediastinal adenopathy (pictures of the CT scan are not available as it was done in another hospital before being referred to us). As part of staging, the patient had a CT scan of the head on November 6, 2022, which was unremarkable. However, staging was completed with a fluorodeoxyglucose (FDG)-positron emission tomography (PET) scan (Figures 1-3) on November 16, 2022, that showed two hypermetabolic left supraclavicular/lower neck nodes, the largest of which is adjacent to the left lobe of the thyroid, measuring 9 mm with standardized uptake value (SUV) of 9.9. Additionally, intense hypermetabolic 5.3 x 2.5 cm left lung mass in the perihilar region, extending into the hilum, was observed with SUV 17.2, along with extensive FDG-avid left hilar adenopathy. Intensely hypermetabolic mediastinal adenopathy involving para-aortic, lower paratracheal, AP window, and subcarinal nodes was seen with SUV up to 9.1. A small left pleural effusion was noticed. Two small, left-sided pleural foci of uptake, one in the apex and the other near the lung base could represent pleural metastases. However, no FDG-avid solid abdominal organ lesions were observed. Multiple intensely hypermetabolic bone lesions are consistent with metastases. They involved the lower cervical spine, several ribs, T8, L2, two sites in the left iliac bone, and a small lesion in the proximal left femur (not a fracture risk). However, the T8 lesion is large, lytic, and possesses a fracture risk.

The patient underwent bronchoscopy on November 21, 2022, during which a biopsy of the left upper lobe was performed. Pathology revealed poorly differentiated adenocarcinoma, consistent with a primary lung tumor. PD-L1 expression was 80%. An ERBB2 c.2313_2324dupATACGTGATGGC mutation (exon 20) was identified, which was also confirmed by a liquid biopsy. When the patient was first seen at the oncology clinic, she was complaining of a dry cough and recent on/off back pain without any urinary or fecal incontinence. She was vitally stable; her weight was 51.8 kg, and her height was 160 cm. She was alert and oriented, not in distress. She had decreased air entry at the left base on auscultation. Abdominal examination was unremarkable; there was no tenderness on spine palpation. Additionally, there was no neurological deficit. Her laboratory findings prior to the first cycle of treatment are presented in Table 1. Notably, both thyroid-stimulating hormone (TSH) and random cortisol levels were within normal ranges. Additionally, her hepatitis serology tests were negative.

**Table 1:** Lab results on January 11, 2023 (prior to first cycle of immunotherapy). N = Normal; H = High; L = Low; LDH: Lactate dehydrogenase; GGT: Gamma-glutamyl transferase; ALT: Alanine aminotransferase; AST: Aspartate aminotransferase.

Laboratory	Patient’s value	Reference values	Units	Note
Hemoglobin	145	120-152	g/l	N
WBC	10.8	4-11	10^9^/L	N
Platelets	427	150-400	10^9^/L	H
Creatinine	67	45-95	umol/L	N
Urea	4.7	3-8	mmol/L	N
Sodium	137	134-144	mmol/L	N
Potassium	3.9	3.5-5.5	mmol/L	N
Chloride	98	98-108	mmol/L	N
Magnesium	0.82	0.70-1.23	mmol/L	N
Corrected calcium	2.52	2.12-2.62	mmol/L	N
Phosphorus	1.01	0.70-1.45	mmol/L	N
Glucose	6.5	3.9-7.7	mmol/L	N
Total bilirubin	4	3-17	umol/L	N
AST	20	15-55	U/L	N
ALT	17	5-40	U/L	N
GGT	54	35-51	U/L	H
Alkaline phosphatase	104	35-145	U/L	N
Albumin	44	35-51	g/L	N
LDH	225	110-220	U/L	H
High-Sensitivity Troponin T	25	0-9	ng/L	H

As per Keynote 024, the patient was started on first-line pembrolizumab. The first cycle began on January 19, 2023. The second cycle started on February 8, 2023. The first two cycles were well tolerated without any side effects. When seen in the clinic prior to her third cycle, the patient complained of palpitations and was tachycardic with a heart rate of 116 beats per minute. The cardiovascular examination revealed no remarkable findings. Her Troponin I level was 19.7 ng/l, and Troponin T had increased to 52 ng/l from 30 ng/l prior to the second cycle. Additionally, the ECG indicated a normal sinus rhythm.

She was admitted to the cardiology division and was started on high-dose steroids (1g IV methylprednisolone) for three days and also started on metoprolol 25 mg orally twice daily. However, the echocardiography results were unremarkable. She showed a good response with decreasing troponin and was switched to oral prednisone with gradual tapering until steroids were stopped and her troponin level was restored to the value before therapy.

After discussion at the tumor board and considering the myocarditis and disease progression, it was decided to switch the patient to second-line therapy with carboplatin/pemetrexed. She received four cycles, with the first on April 14, 2023, and the fourth on June 16, 2023. The patient demonstrated a good response after two cycles and stable disease after four cycles. However, due to hematological toxicity, carboplatin was discontinued, and she was switched to maintenance pemetrexed starting July 7, 2023. The fifth maintenance cycle was administered on August 29, 2023. The last CT in September 2023 showed a new left para-aortic abdominal adenopathy, suspicious for metastasis. Mild improvement of pulmonary parenchymal densities was observed along the left major fissure and medial right lower lobe with stable-appearing bone lesions.

Notably, the patient received 16 Gy of radiation in two fractions to T8 in December 2023 due to a compression fracture, although the MRI did not show any cord compression. In March 2023, she received two fractions of radiation to the iliac bone for a lytic lesion with a probable pathologic incomplete fracture. Additionally, in May 2023, the patient underwent injection laryngoplasty for vocal cord paralysis and responded well. She also experienced an allergic skin reaction to Septra.

## Discussion

ICI-induced myocarditis

The incidence of ICI-induced myocarditis ranges from 0.06% to 1%, according to different trials, and is found to be higher in patients treated with double immunotherapy compared to single-agent immunotherapy [1-2]. 

Its timing is usually in the first three months of starting ICI [3]. However, it can happen at any time, as shown in a case report that showed ICI-induced myocarditis after two years of ICI [4]. 

The most important risk factor for developing ICI-induced myocarditis is the presence of underlying autoimmune disease [5]. Other risk factors include pre‐existing cardiac disease and diabetes mellitus. 

The presentation of ICI-induced myocarditis ranges from asymptomatic abnormal cardiac enzymes to heart failure and death. Follow-up for suspected ICI-induced myocarditis should include at least ECG, serial troponin, brain natriuretic peptide (BNP), creatine phosphokinase (CPK) (to rule out concurrent myositis), echocardiogram, and chest X-ray. Cardiology should be consulted, and further investigation should be guided by cardiology, such as stress tests, cardiac catheterization, cardiac MRIs, and sometimes a cardiac biopsy. If suspicion of myositis or myasthenia is raised, the patient should be referred to rheumatology and neurology, respectively. 

Grading of ICI-induced myocarditis according to the American Society of Clinical Oncology (ASCO) [6]: Grade 1 = abnormal cardiac biomarker testing without symptoms and with no ECG abnormalities; Grade 2 = abnormal cardiac biomarker testing with mild symptoms or new ECG abnormalities without conduction delay; Grade 3 = abnormal cardiac biomarker testing with either moderate symptoms or new conduction delay; Grade 4 = moderate to severe decompensation, IV medication or intervention required, life-threatening conditions. Patients need to be admitted to the CCU and put on a cardiac monitor. The first line of treatment includes high-dose steroids, usually 1 g of IV methylprednisolone for three days. The time of initiating the steroids is crucial, as shown in a retrospective study [7]. In this study, patients were stratified based on the dose of methylprednisolone received: low (<60 mg/day), intermediate (60-500 mg/day), and high (501-1,000 mg/day). The findings indicated that patients who received steroids within 24 hours, regardless of the dosage, had the best outcomes, and patients receiving steroids after 72 hours, regardless of dosage, showed the worst outcome. The same study showed that patients receiving high doses of steroids had a 73% lower risk of major adverse cardiac events. As per the European Society for Medical Oncology guidelines (ESMO), the following step in management depends on the response to high-dose steroids. If troponin is reduced by >50% from the peak, then we switch to 1mg/Kg of oral prednisolone with a weekly reduction of 10mg over 4-6 weeks. On the other hand, if the patient is refractory to high-dose steroids, we continue IV steroids and add a second-line immunosuppressive drug (tocilizumab 8mg/kg or mycophenolate mofetil). The third line would include alemtuzumab or abatacept. Furthermore, IV immunoglobulin and plasma exchange should be considered for hemodynamically unstable patients [8]. Serial troponin monitoring during ICI is a debatable issue. Some hospitals check troponin levels as part of the routine blood workup before every cycle of ICI. The schools that practice this approach found it to be a non-invasive biomarker to monitor cardiotoxicity and to avoid underdiagnosing asymptomatic ICI-induced myocarditis [9]. Elevated troponin can be attributed to factors other than myocarditis. Additionally, treating elevated troponin as ICI-induced myocarditis could result in an unnecessary stoppage of ICI, leading to cancer progression. It would be preferable to monitor serial troponin, but with careful interpretation of the results [10]. The restarting of ICI should be discussed at multidisciplinary rounds. This depends on many factors, such as the severity of toxicity, recovery from toxicity, single or double immunotherapy, and disease control.

HER2 alterations in NSCLC

HER2 alterations in NSCLC, with an incidence of 1%-3% [11-12], are characterized by either gene mutation, gene amplification, or protein overexpression [11]. The most common alterations include insertions in exon 20, but point mutations have also been reported. The most significant advancement in treating advanced or metastatic HER2 NSCLC is trastuzumab deruxtecan, an antibody-drug conjugate, as shown in Destiny-Lung's phase 2 clinical trial [13]. This treatment has shown an objective response rate of 55%, with a median duration of response of 9.3 months. The median progression-free survival is recorded at 8.2 months, and the median overall survival at 17.8 months. Common side effects include neutropenia and interstitial lung disease. Furthermore, the efficacy of combining trastuzumab with other chemotherapy agents is currently under investigation.

Trastuzumab and cardiac toxicity 

The clinical manifestation of trastuzumab’s cardiac toxicity is decreased left ventricular ejection fraction (LVEF) and, less commonly, heart failure.

The incidence of cardiotoxicity when trastuzumab is administered alongside anthracyclines ranges from 4.0% to 18.6% for decreased LVEF and from 0.4% to 4.1% for severe heart failure. Conversely, when trastuzumab is given without anthracyclines, the incidence of LVEF decline and symptomatic heart failure is 3.2% and 0.5%, respectively [14]. Importantly, trastuzumab-related cardiotoxicity is not associated with cumulative dosage and is often reversible upon treatment discontinuation. Additionally, patients often tolerate a rechallenge with trastuzumab after recovery [15].

Risk factors for higher incidence include age greater than 50 years, previous or concurrent anthracycline, obesity, pre-existing cardiac dysfunction, and hypertension. On the other hand, valvular heart disease, coronary artery disease, and concurrent radiation in adjuvant sitting did not show increased risk.

Patients who plan to start trastuzumab-based therapy should have a cardiac baseline evaluation, including an echocardiogram. The surveillance for trastuzumab-related cardiac toxicity in the adjuvant setting usually involves repeated evaluations for cardiac function at 3, 6, 9, and 12 months. In metastatic disease, LVEF is monitored at baseline and then only in the presence of symptoms. Guidelines for the management of trastuzumab-related cardiac toxicity usually follow clinical trials of adjuvant trastuzumab (the NSABP B-31 and NCCTG N9831 trials) [16].

## Conclusions

ICI-induced myocarditis is a serious complication. We need to conduct prospective trials to manage irAEs, as most of the guidelines are based on clinical experience. Thus, we need prospective trials to address the issue of serial troponin monitoring and interpret the values. Additionally, there is a need for trials aimed at advancing targeted therapy for HER2 alterations in NSCLC from later lines of treatment to the first line.
